# Cell Staining Microgels Derived from a Natural Phenolic Dye: Hematoxylin Has Intriguing Biomedical Potential

**DOI:** 10.3390/pharmaceutics16010147

**Published:** 2024-01-22

**Authors:** Mehtap Sahiner, Aydin K. Sunol, Nurettin Sahiner

**Affiliations:** 1 Department of Bioengineering, Faculty of Engineering, Canakkale Onsekiz Mart University Terzioglu Campus, Canakkale 17100, Turkey; msahiner@comu.edu.tr; 2 Department of Chemical & Biomedical Engineering, Materials Science and Engineering Program, University of South Florida, Tampa, FL 33620, USA; asunol@usf.edu; 3 Department of Chemistry, Faculty of Sciences & Arts, and Nanoscience and Technology Research and Application Center (NANORAC), Canakkale Onsekiz Mart University Terzioglu Campus, Canakkale 17100, Turkey; 4 Department of Ophthalmology, Morsani College of Medicine, University of South Florida Eye Institute, 12901 Bruce B Down Blvd, MDC 21, Tampa, FL 33612, USA

**Keywords:** natural phenolic particles, hematoxylin microgels, fluorescent, antioxidant, biocompatible microgels, α-glucosidase inhibitor

## Abstract

Hematoxylin (HT) as a natural phenolic dye compound is generally used together with eosin (E) dye as H&E in the histological staining of tissues. Here, we report for the first time the polymeric particle preparation from HT as poly(Hematoxylin) ((p(HT)) microgels via microemulsion method in a one-step using a benign crosslinker, glycerol diglycidyl ether (GDE). P(HT) microgels are about 10 µm and spherical in shape with a zeta potential value of −34.6 ± 2.8 mV and an isoelectric point (IEP) of pH 1.79. Interestingly, fluorescence properties of HT molecules were retained upon microgel formation, e.g., the fluorescence emission intensity of p(HT) at 343 nm was about 2.8 times less than that of the HT molecule at λ_ex_: 300 nm. P(HT) microgels are hydrolytically degradable and can be controlled by using an amount of crosslinker, GDE, e.g., about 40%, 20%, and 10% of p(HT) microgels was degraded in 15 days in aqueous environments for the microgels prepared at 100, 200, and 300% mole ratios of GDE to HT, respectively. Interestingly, HT molecules at 1000 mg/mL showed 22.7 + 0.4% cell viability whereas the p(HT) microgels exhibited a cell viability of 94.3 + 7.2% against fibroblast cells. Furthermore, even at 2000 mg/mL concentrations of HT and p(HT), the inhibition% of α-glucosidase enzyme were measured as 93.2 ± 0.3 and 81.3 ± 6.3%, respectively at a 0.03 unit/mL enzyme concentration, establishing some potential application of p(HT) microgels for neurogenerative diseases. Moreover, p(HT) microgels showed two times higher MBC values than HT molecules, e.g., 5.0 versus 2.5 mg/mL MIC values against Gram-negative *E*. *coli* and Gram-positive *S. aureus*, respectively.

## 1. Introduction

Hematoxylin (HT) is a plant-derived phenolic compound (from the logwood tree) [1], and it was first discovered by Spanish explorers in the Yucatan peninsula (Mexico) in 1502 [2]. It is used as a dyeing agent in some industries such as leather, textiles, and even hair dyeing. It has also been widely used in histopathology to stain various cells and tissues since the 1800s [3,4,5]. HT is composed of two benzene rings and four aromatic hydroxyl groups that do not stain directly. The staining occurs when it is oxidized to hematein. HT salts with different oxidation states of metal ions of Al, Cr, Cu, and Fe metal are used to dye textile materials in various colors, e.g., ranging from black to purple and violet. After discovering the HT dyeing characteristics, histologists have developed several different mordants [2]. Mordants are mineral salts that aid the dye to bind better to the material to be dyed. HT is also used to visualize prostate, bladder, and mount cancer for imaging in biomedical research laboratories [6,7].

It has been reported that upon the loading of metal ions such as Fe(III), Gd(III), and Zr(III) onto particles derived from polyphenol as well as fluorescent dyes, these materials can be used in positron emission tomography, single photon emission computed tomography, magnetic resonance, near-infrared, fluorescence, and bioimaging applications [8]. It has been reported that Bi(III) ions complexes with HT can be used in the determination of the arginine amino acid [9]. There are studies on the cell nucleus and DNA staining of the Al(III)-HT complex [10]. There is even a report on the use of HT in the diagnosis of pancreatic cancer, breast cancer, and skin cancer [11,12,13]. It is stated that ATR-FTIR spectroscopy could be used as a complementary method to the microscopic examination of HT and eosin-stained tissues in diagnosing lung cancer [14]. The interest in flavonoid-based materials is increasing steadily due to its antimicrobial, antioxidant, and anti-cancer properties. In a study, it was also reported that HT prevents amyloid β-protein fibrillation, which is important in the treatment of Alzheimer’s disease, alleviates amyloid-induced cytotoxicity, and can be used as a therapeutic agent in the treatment of Alzheimer’s disease [15]. It was pointed out that HT can be a protein tyrosine kinase inhibitor and thus can be used in cancer treatment [16].

In advanced organisms (eukaryotic cells), the cell nucleus is a specialized organelle and very hard to deliver active agents to it. To treat various disorders, including cancers, bioactive substance nanocarriers are designed to deliver bioactive molecules to the nucleus, which is considered to be the ultimate target. Nanocarriers increase the permeability and retention of drugs in tumors [17]. Recently, nanocarrier systems have been mainly used to transport bioactive molecules to cancer cells in order to target nuclei that are typically difficult to reach. Nanocarriers with degradation and cell staining ability can offer additional advantages to monitor the carried active agent interactions with the diseased tissues and cells. Therefore, nano- and microcarriers derived from natural phenolic compounds such as HT can be used for dual purposes, e.g., as a controlled drug/active agent delivery device and for bioimaging utilizing hydrolytic degradation.

In this study, HT microgels as (p(HT)) were synthesized via a simultaneous polymerization and crosslinking method in a single step via a microemulsion method for the first time. The characterization of p(HT) microgels was performed using FT-IR, thermal gravimetric analyzer (TGA), scanning electron microscope (SEM), zeta potential measurements, and fluorescence spectroscopy analysis. Hydrolytic degradation of p(HT) microgels prepared at different HT to crosslinker ratios (GDE) was investigated at different pHs. Moreover, antioxidant properties, cytotoxicity on fibroblast cells, and α-glucosidase enzyme interactions of HT and p(HT) microgels were examined.

## 2. Materials and Methods

### 2.1. Materials

Hematoxylin crystalline (85%, Fisher chemical, Hampton, NH, USA) and glycerol diglicydyl ether (GDE, technical grade, Sigma, St. Louis, MO, USA) were used as the monomer and crosslinker, respectively. L-alpha-lecithin, (granular, from soybean oil, Thermo scientific, Waltham, MA, USA) as the surfactant and 2,2,4-trimethylpentane (isooctane, Thermo scientific 99+%, Sigma) as the solvent were used as received in p(HT) microgels preparation.

Folin and Ciocalteau’s phenol reagent (FC, Sigma-Aldrich, St. Louis, MO, USA), sodium nitrite (Merck, extra pure, Rahway, NJ, USA), aluminum chloride (Merck, anhydrous powder sublimed from synthesis), gallic acid (GA, 97.5–102.5%, Aldrich, St. Louis, MO, USA), and rosmarinic acid (RA, 96%, Aldrich) were employed in antioxidant assays.

For cell viability studies, L929 fibroblasts (mouse connective tissue) were employed with Dulbecco’s Modified Eagle’s Medium (DMEM, Pan BioNTech, Aiden Bach, Germany), containing 4.5 g/L glucose and 3.7 g/L sodium pyruvate, while 0.5 g/mL L-Glutamine was used as a cell culture medium. Fetal bovine serum (FBS inactivated, Pan BioNTech, Aiden Bach, Germany) and Ca/Mg-free trypsin/EDTA (Pan BioNTech, Aiden Bach, Germany) were used as received. MTT agent, 3-(4,5-dimethylthiazol-2-yl)-2,5-diphenyltetrazolium bromide (neo-Roxx, Einhausen, Germany) was used as received. Trypan blue solution (0.5%, Biological Industries, Bet-Haemek, Israel) and dimethyl sulfoxide (DMSO, 99.9%, Carlo-Erba, Val-de-Reuil, France) were used for cell counting and dissolving the formazan crystals. 

α-Glucosidase from *Saccharomyces cerevisiae* (100 unit/mg, Sigma-Aldrich, St. Louis, MO, USA) and 4-nitrophenyl-α-D-glucopyranose (99%, Acros Organics, Geel, Belgium) were used as the enzyme and substrate.

For antibacterial activity tests, a Gram-negative bacterium, *E. coli* (ATCC 8739), and a Gram-positive bacterium, *S. aureus* (ATCC 6538), were obtained from KWIK-STIK™ Microbiologics (St. Cloud, MN, USA). Nutrient agar (NA) and nutrient broth (NB) as growth media for bacteria studies were procured from BD DifcoTM (Becton, Dickinson and Company, Sparks, MD, USA) and used as received.

### 2.2. Poly(hematoxylin) Microgel Synthesis and Characterization

To prepare p(HT) microgels, 0.1 M 30 mL lecithin in isooctane as the surfactant solution was employed as the microemulsion system to prepare p(HT) microgels in accordance with the literature with slight modification [18]. In short, 0.2 g HT was dissolved in 1 mL of 1 M NaOH solution. Then, 1 mL of HT solution was put into 30 mL of 0.1 M lecithin/isooctane solution while stirring at 1000 rpm. Next, 100, 200, and 300% mol of GDE (based on HT amount) were added as a crosslinking agent, separately in the prepared separate HT solutions, respectively. Microgels were obtained by mixing them at room temperature for 24 h and centrifuging them at 10,000 rpm for 10 min. To remove the surfactant and un-reacted species, e.g., HT and crosslinker, p(HT) microgels were washed with isooctane once, with ethanol twice, and with ethanol:water mixture (1:1 volume) twice by repeated centrifugations. Finally, the p(HT) microgels were re-washed with acetone, dried with a heat gun, and dried further in a vacuum oven for further use. 

The p(HT) microgels were imaged using scanning electron microscopy (SEM, Hitachi Ultra High-Resolution Analytical FE-SEM SU-70, Tokyo, Japan). To acquire SEM images, p(HT) microgels were coated with gold in a vacuum, and the SEM images were attained at 7–25 kV operating voltage. 

FT-IR spectroscopy (Nicolet IS10, Thermo, Waltham, MA, USA) was used to analyze the functional group of HT and p(HT) particles in the spectral range of 4000–650 cm^−1^. Thermal analyses of p(HT) microgels were performed using an SII TG/DTA 6300 thermogravimetric analyzer (Tokyo, Japan). In TGA, a ceramic pan holding approximately 3–5 mg of p(HT) microgels was heated from 50 to 500 °C, under a N_2_ flow of 100 mL min^−1^ at a 10 °C/min heating rate, and the weight loss# versus T was recorded. The functional groups of HT and p(HT) microgels were also assessed with a Zeta Potential Analyzer (Zeta-Pals, Brookhaven, NY, USA) to measure the zeta potentials of the p(HT) particle suspension in 1 mM KNO_3_ solutions at 1.0 mg/mL concentrations. A surface charge measurement of the p(HT) microgel structure at different pH values was also used to determine the isoelectric points (IEP). Photoluminescence properties of HT and p(HT) were evaluated using a Fluorescence Spectrometer (Lumina, Thermo-Scientific, Boston, MA, USA) instrument in the spectral range of 270–540 nm wavelength.

Two antioxidant tests were performed on HT and p(HT) microgels. One of which is the total phenol content (TPC) [19]. For TPC tests, 1000 µg/mL of HT molecule and p(HT) microgels were prepared and the particle suspension solutions were allowed to undergo hydrolytic degradation in a shaking bath in PBS at 37.5 °C for 5 days. The antioxidants of the degradation products were tested depending on the time and concentration. The suspension solution was diluted several times, between 500 and 62.5 µg/mL. A 96-well plate was filled with 20 µL of sample solution and 125 µL of FC solution. After adding 100 µL of 0.7 M Na_2_CO_3_ aqueous solution, the mixture was then left to sit in the dark for two hours for incubation. After that, the absorption value of the solutions was read at 760 nm with a microplate reader (Thermo Scientific, Multiskan SKY, Waltham, MA, USA). Gallic acid (GA) was used as a standard material and the results were given as GA equivalents.

The other method used in the determination of antioxidant properties of HT molecules and p(HT) particles was the total flavonoid content (TFC) assay. For this purpose, 50 µL of the suspended solution in the 1000–62.5 µg/mL concentration range of HT and p(HT) was added to 96 wells, followed by the addition of 25 µL of 3% NaNO_2_ solution. Then, the 6% AlCl_2_ solution was put into these wells. Finally, 100 µL of a 1 M NaOH solution was added to the wells. At 405 nm, the absorbance values of the solutions were compared to a previously generated calibration of TPC values of rosmarinic acid (RA), and the results were expressed in µg/mL RA equivalences of total phenol.

The biocompatibility of HT-based materials was evaluated using the MTT cell viability assay as described by Mosmann [20]. In the cytotoxicity studies, a laminar airflow biosafety cabinet was used to gently thaw frozen L929 fibroblasts at 37 °C. Next, the thawed cells were placed in DMEM with 10% FBS and centrifuged at 100× *g* for 5 min. Then, the incubation was carried out for a few days at 37 °C in a CO_2_ incubator (5% CO_2_/95% air atmosphere) after the supernatant was removed and 1 mL of fresh DMEM was added. As the desired confluence of cells was achieved, trypsin-EDTA was used for 5 min to detach the cells. Following the isolation of the cells, a detachment procedure was performed, resulting in a suspension. The suspension was then subjected to centrifugation at 100× *g*. Subsequently, the cells were enumerated using a hemocytometer. 

A total of 20 mg of HT and p(HT) microgels were separately weighed and suspended in 10 mL of DMEM to obtain an initial concentration of 2 mg/mL. DMEM was then used to dilute these samples to 1000–50 µg/mL concentrations. Following a cell count, L929 fibroblasts were seeded into a 96-well plate with a volume of 100 μL of cell suspension, containing approximately 5 × 10^3^ cells/mL. The plate was then incubated for 20 h at 37 °C within a CO_2_ incubator to allow cell growth and proliferation. Then, the old media were discarded, and 0.1 mL of HT-based microgels were placed onto the wells and the plate was incubated for 24 h. After removing the media, the wells were washed with PBS solution. The wells were then filled with 100 μL of a 0.5 mg/mL MTT solution prepared in DMEM and kept in the dark for 3 h. Following this, 200 μL of DMSO was added to dissolve the formazan crystals, which were thoroughly mixed and left to settle for 20 min. The optical density was then measured at 590 nm using a microplate reader (Multiskan GO, Microplate Photometer, Thermo Fisher Scientific, Waltham, MA, USA).

The enzyme, α-glucosidase was used as a model enzyme, and a 4-nitrophenyl-α-D-glucopyranose was used as a substrate in the enzyme inhibition studies. The experiment was conducted based on the literature [21]. In short, sample solutions of different concentrations are placed in 96 wells as 70 µL 0.03 unit enzyme/mL is added to it. The first reading is made at 405 nm. After 5 min, 70 µL of substrate solution is added. After 20 min, the final reading is taken, the necessary calculations are made, and the % inhibition value is calculated from the formula below:

Inhibition% = (1 − (A_sample_/A_control_)) × 100

When A_control_ is measured without added HT monomer or p(HT) eluate, A_sample_ is measured with the added monomer or eluate. 

The time-dependent degradation of p(HT) microgels prepared at different amounts of crosslinkers was studied in a PBS environment at 37.5 °C. Additionally, the degradation of p(HT) microgel with time prepared at 300 moles % crosslinker (300×) at three different pH media (pH 7.4 phosphate buffer, pH5.4 citrate buffer, and pH 9 borate buffer) was studied at 37 °C. For each buffer solution, HT calibration was used and prepared via a UV-Vis spectrophotometer at 290 nm to assess the degradation amounts of HT from p(HT) microgels.

Antimicrobial capabilities of HT and p(HT) microgels at 100× were determined via broth micro-dilution test against *E. coli* (ATCC 8739) and *S. aureus* (ATCC 6538) stains according to the literature [22]. Briefly, samples were suspended in a 0.9% saline solution at 20 mg/mL initial concentration, and the concentrations ranged from 10 mg/mL to 0.02 mg/mL. Sample solutions at varying concentrations were inoculated in nutrient broth medium in 96 wells and incubated for 24 h. At the end of the incubation, minimum inhibitory concentration (MIC) and minimum bactericidal concentration (MBC) were determined according to the literature [22]. Gentamicin sulfate was used as a positive control. 

## 3. Results

### 3.1. Characterization of p(HT) Microgels

**Figure 1:**
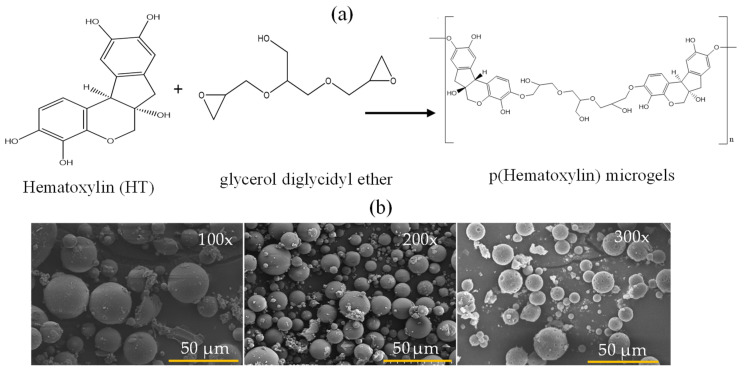
(**a**) Schematic presentation of p(HT) microgel preparation from the related molecules and (**b**) SEM images of p(HT) microgels prepared at three different crosslinker ratios (100×, 200×, and 300×), respectively.

It is very well known that microemulsion polymerization and/or the crosslinking technique is widely used to prepare particles or microgels employing different surfactants for different monomers or polymers [23,24]. Microgels of HT as p(HT) were synthesized by the crosslinking of HT monomer units with GDE as crosslinker at 100, 200, and 300% mole ratios of HT via the microemulsion method in a single step. The p(HT) microgels prepared at 300% GDE resulted in better particle formation and higher yields, >70% gravimetrically, in comparison to the other amounts of crosslinker ratios. The schematic representation of the p(HT) microgel preparation is shown in Figure 1a. The prepared p(HT) microgels are in the 10–30 micrometer size range as shown in Figure 1b via the SEM images with spherical shapes. In Figure 1b, the SEM images of p(HT) microgels prepared at three different crosslinker % ratios to HT as 100×, 200×, and 300× were given. The average size of p(HT) microgels was determined using SEM images according to the Image J (V. 1.8.0) software program. Accordingly, the average value of p(HT) 100× microgels was 20.3 + 7.2 μm, the average value p(HT) 200× microgels was 17.9 + 9.0 μm, and the average value p(HT) 300× was found to be 9.3 + 4.1 μm. As the crosslinker ratio increased, smaller-sized p(HT) microgels were obtained. Interestingly, p(HT) microgels prepared at 300× have some pore features on their surfaces and this will be the subject of another paper.

**Figure 2:**
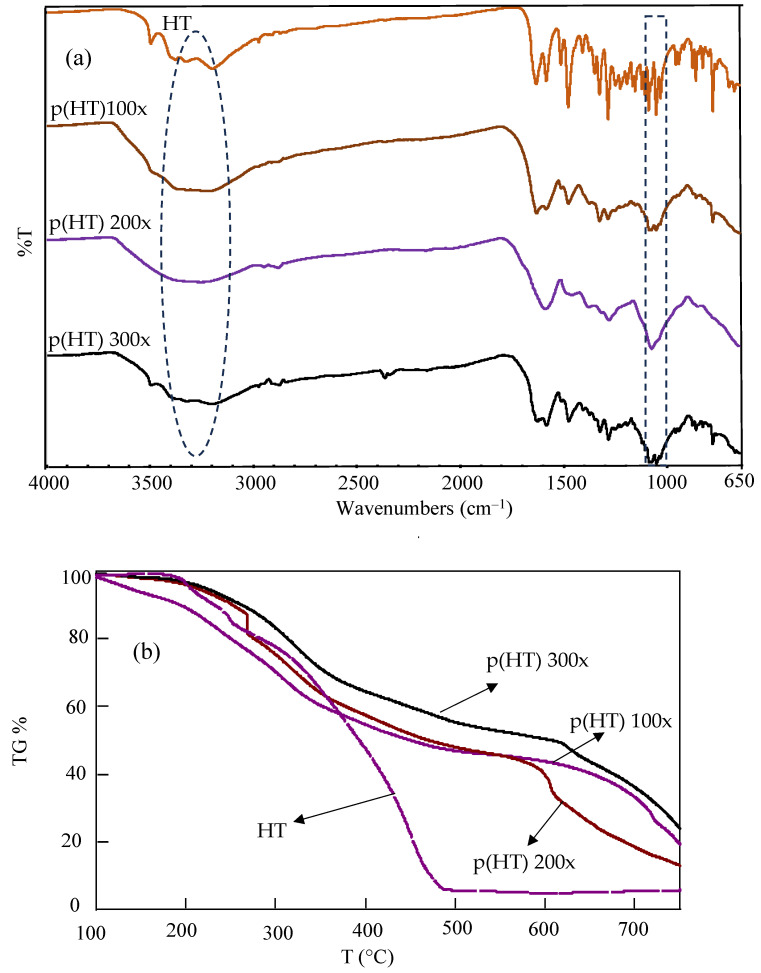
(**a**) FT-IR spectra and (**b**) TG analysis of HT and p(HT) microgels.

The FT-IR spectra and TG analysis of HT and p(HT) microgels are given in Figure 2a and Figure 2b, respectively. According to the FT-IR spectrum in Figure 2b, HT is successfully crosslinked with GDE as can be seen by the formation of -CH_2_-O-R of the ether bond at the 1050 cm^−1^ wavelength. The -OH peak for HT is seen in the range of 3393–3175 cm^−1^. The same peaks are also seen in the FT-IR spectra of all p(HT) microgels prepared at different crosslinker ratios. However, the peak intensities widen due to the crosslinking of -OH groups with the epoxide groups of the crosslinker, GDE, and newly formed -OH groups that are not linked to the phenolic unit as shown in Figure 1a.

Thermal gravimetric analysis of the HT and p(HT) microgels was carried out in the range of 100–750 °C. The TGA result given in Figure 2b illustrates that the HT molecule degraded at about 3 wt% at 187 °C, 17 wt% at 253 °C, and 94 wt% at 486 °C. At 750 °C, the remaining HT was 5.7%, whereas for p(HT) microgels crosslinked at 100×, 200×, and 300×, the remaining HT was 19.3%, 13.0%, and 23.8%, respectively. The TG analysis results show that p(HT) particles are thermally more stable than HT molecules at higher temperatures even though both show more or less similar degradation profiles between 100 and 400 °C.

The zeta potentials of p(HT) microgels prepared at 100× against different solution pHs were measured to determine the isoelectric point (IEP). Initially, p(HT) microgels at 1 mg/mL were placed in a 1 mM 3 mL KNO_3_ solution and the measured pH was 10.21 with a zeta potential value of −34.6 mV.

**Figure 3:**
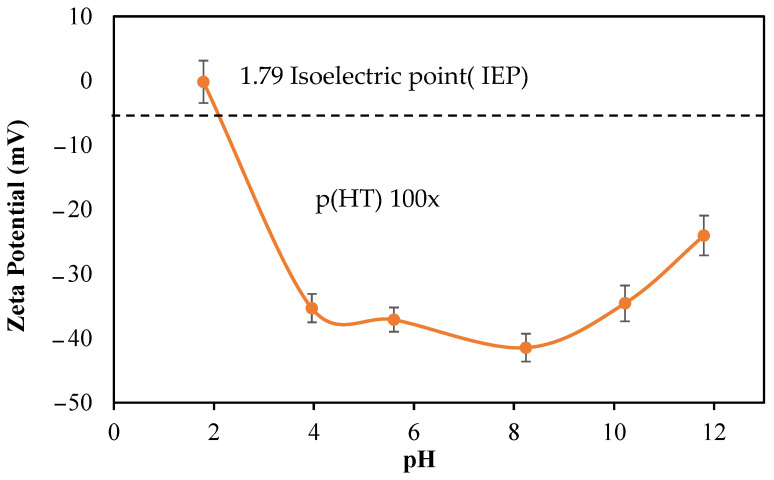
Zeta potential values of p(HT) microgel suspended at different solution pHs at 1.0 mg/mL.

As illustrated in Figure 3, the zeta potentials of p(HT) microgels at different pHs are measured and the IEP of p(HT) microgels is calculated as 1.79. So, at this pH (1.79), the p(HT) particles have a net zero charge stating that at about neutral pHs, 7, of p(HT) microgels are negatively charged, about −40 mV and are stable in aqueous environments. 

**Figure 4:**
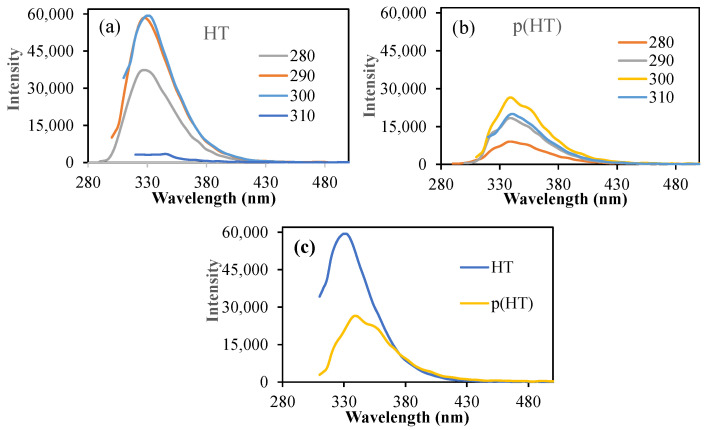
(**a**) The fluorescence spectra of (**a**) HT and (**b**) p(HT) microgels at different excitation wavelengths; (**c**) the fluorescence spectra of HT and p(HT) microgels at 300 excitation wavelengths.

As the HT molecule has photoluminescence characteristics, the fluorescent spectroscopic analysis of HT and p(HT) microgels was carried out using fluorescent spectroscopy, and the corresponding spectrums are given in Figure 4. 

As can be seen, HT molecules have very high intensity at an excitation wavelength range of 280–300 nm excitation with an emission wavelength of 330 nm as seen in Figure 4a. In the literature, it was mentioned that a fluorescence microscopy technique using H&E-stained sections is an easy, reliable, and inexpensive method that can be used to evaluate intact and pathological teeth for morphological characteristics [25]. Also, the fluorescence properties of H&E can be used to assess elastic tissue, basement membrane zone thickening, and dermatophytes on routinely processed and stained sections, without waiting for special stains to develop or requiring special handling [26]. On the other hand, p(HT) microgels showed somewhat reduced emission intensities upon executions at the same wavelength range as shown in Figure 4b. The highest emission intensity of 26,388 (a.u) at about 340 nm upon excitation at 300 nm excitations was almost half of the HT molecules (intensity 59,320 a.u) at the same emission and excitation conditions as illustrated in Figure 4c. This undoubtedly highlights that p(HT) particles retain the fluorescence property of HT molecules. Therefore, p(HT) microgels can be used not only as photoactive materials but also as cell staining drug carriers.

### 3.2. Bioactive Properties of p(HT) Microgels

**Figure 5:**
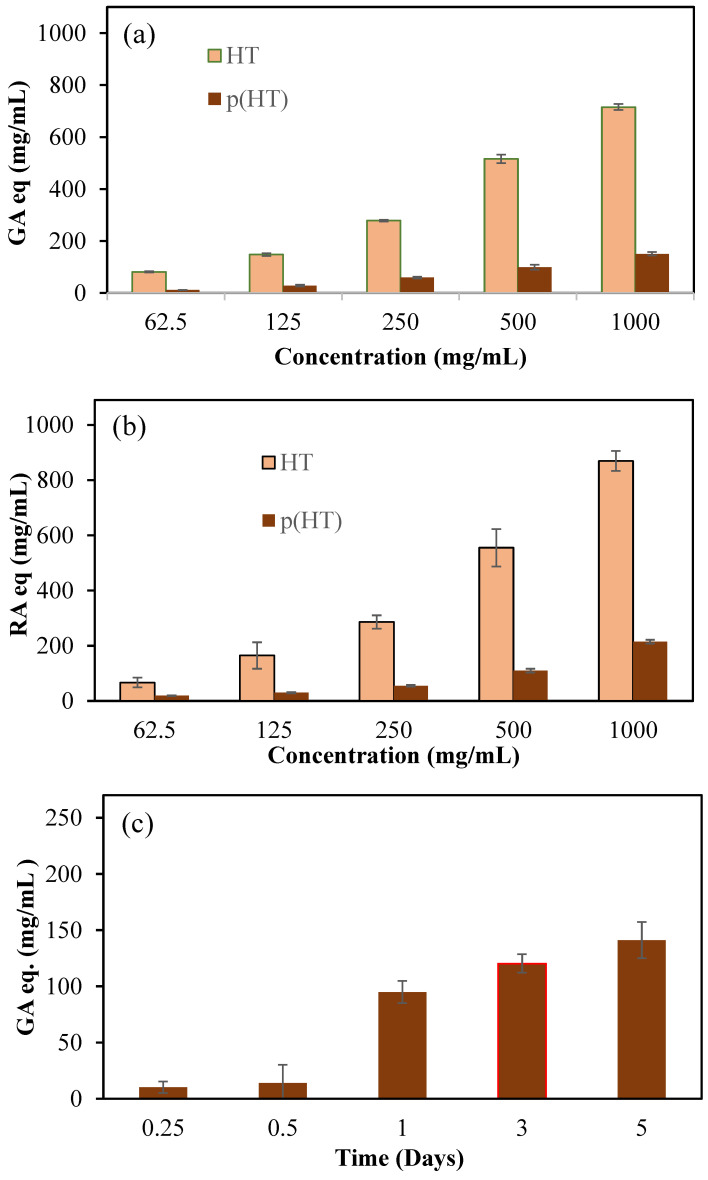
(**a**) Total phenol content (TPC) GA equivalency of HT and p(HT) particles at different concentrations, (**b**) total flavonoid content as rosmarinic (RA) equivalency (RA eq) for HT and (HT) particles at different concentrations, and (**c**) time-dependent antioxidant values of degraded products of p(HT) microgels via the TPC test.

Phenolic compounds are a group of secondary metabolites found only in plants. Aromatic rings substituted with hydroxyl groups are the basic element of their chemical structure. Due to their reducing properties, phenolic compounds are effective antioxidant materials because they act as hydrogen donors and scavengers of OH• and O•_2_ radicals [27]. Many fruits and vegetables are known for their antioxidant properties, and it is even reported that extracts from the flowers of the logwood tree have antioxidant properties in in vitro studies [28]. Total phenol content (TPC) and total flavonoid content (TFC) assays are commonly used to assess the antioxidant values of phenolic compounds. Therefore, these tests were applied to HT and hydrolytically degraded (HT) microgels at concentrations ranging from 62.5 to 1000 mg/mL, and the corresponding results in terms of gallic acid (GA equivalent) and rosmarinic acid equivalence (RA equivalent) are given in Figure 5a and Figure 5b, respectively. 

According to the results of both test methods, the antioxidant capacity of HT molecules in an increasing concentration dependence can also be seen in p(HT) microgels to a lesser degree. Keeping in mind that p(HT) particles are degradable, the degradation products are HT molecules, and the degree of degradation depends on the amount of crosslinker used. According to the TPC test method, 1000 mg/mL of HT showed 715.8 ± 11.6 GA equivalent antioxidant capability and, at the same concentrations, p(HT) microgels showed antioxidant activity of 150.8 ± 7.6 mg/mL GA eq. In the TFC test, the same concentration of HT was displayed to have 819.1 ± 36.3 RA equivalent antioxidant capability whereas p(HT) microgels possessed lower antioxidant ability at 215.2 ± 7.01 mg/mL RA eq. Also, the antioxidant values of the solutions of p(HT) microgels at different times were determined via the TPC test and the corresponding graph is presented in Figure 5c. As can be seen from Figure 5c, as the degradation time increases, the TPC values increases as expected since the degrading amount of HT molecules increases with time. The antioxidant ability of p(HA) microgels along with the fluorescence properties render p(HA) microgels many advantages in biomedical applications. For example, innate antioxidant materials with the capability of long-term cell staining can allow for the visualization of cell proliferation or afford monitoring of the development of a certain duration of time of diseased tissues and can thus be used for diagnostic purposes as an application in the prognosis of diseases or in the differentiation of cells for possible tissue engineering applications. 

**Figure 6:**
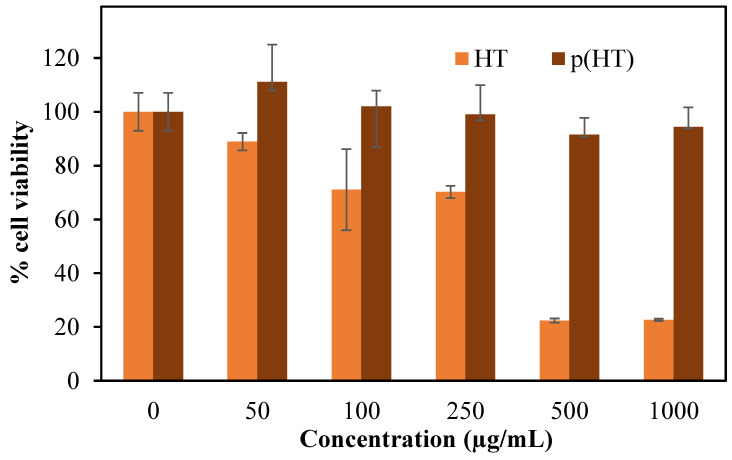
Cytotoxicity of HT and p(HT) microgels on L929 fibroblasts at 50,100, 250, 500, and 1000 µg/mL concentrations at 24 h incubation time.

The cytotoxicity of HT and p(HT) microgels were also examined by incubating these materials for a 24 h incubation period against L929 fibroblast cells at different concentrations ranging from 50 to 1000 g/mL, and the results are summarized as shown in Figure 6.

As seen in Figure 6, p(HT) microgels are more biocompatible than HT molecules and even at 1000 mg/mL concentration, p(HT) microgels demonstrated 94.3 + 7.2% cell viability in comparison to HT molecules which exhibited 22.7 + 0.4% cell viability at the same concentration. These results establish that the p(HT) molecule has great potential for in vivo biomedical applications in processing all the properties of HT molecules. 

**Figure 7:**
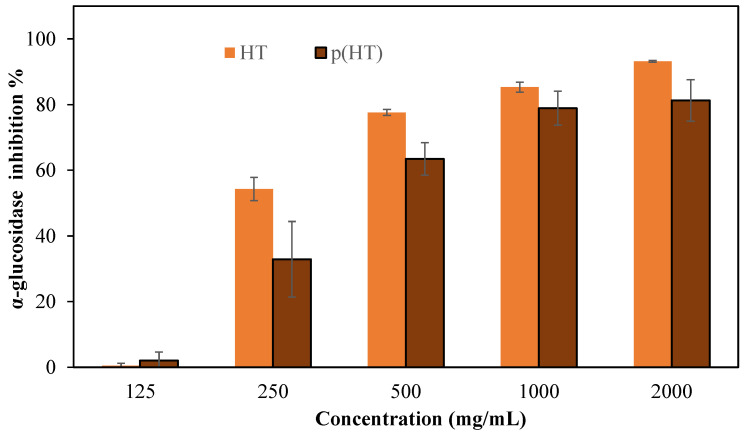
The % inhibition values of the α-glucosidase enzyme of HT and p(HT) at 125–2000 mg/mL concentrations in a pH 6.9 phosphate buffer solution.

Enzyme inhibitors such as α-glucosidase inhibitors are used in the treatment of type II diabetes because they prevent the breakdown of disaccharides and polysaccharides [29]. Here, α-glucosidase inhibition of HT and p(HT) microgels was studied at a concentration range of 125–2000 mg/mL and the corresponding results are shown in Figure 7. HT molecules inhibited 93.2 ± 0.3% of the enzyme at 2000 mg/mL concentration, and, at the same concentration, the p(HT) microgels were capable of inhibiting 81.3 ± 6.3% of the enzyme and thus corroborate the further versatility of p(HT) microgel in diverse biomedical use.

It is stated that the *Caesalpinia sappan Linn*. plant, which contains abundant hematoxylin, is used as a traditional treatment for diabetes diseases [30]. It is also reported that Haematoxylon campechianum extract inhibits cyclooxygenase [28]. The α-glucosidase enzyme inhibition results agree with the literature. These findings suggest that HT and p(HT) microgels possess meaningful potential to be used as effective inhibitors of the different enzymes. Plant materials in their raw form contain a diverse range of active ingredients possessing highly useful therapeutics. Diabetes mellitus is a complex disease that requires persistent treatment avenues. Therefore, materials such as p(HT) cannot entirely replace conventional pharmacotherapy, but they can help in reducing the dose or number of drugs needed to control blood sugar levels [31].

**Figure 8:**
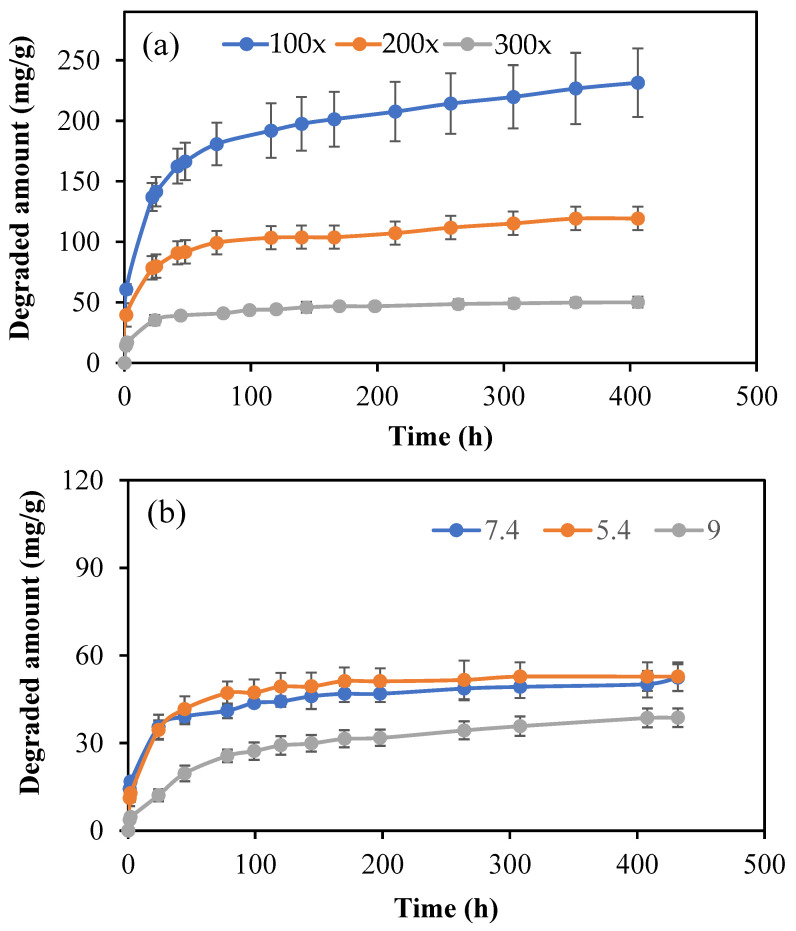
(**a**) Hydrolytic degradation profile of p(HT) microgels prepared at 100×, 200×, and 300× in PBS at pH 7.4 and 37 °C and (**b**) the hydrolytic degradation of p(HT) microgels prepared at 300× in pH 7.4 phosphate buffer, pH 5.4 citrate buffer, and pH 9 borate buffer.

As HT molecules are antioxidant and possess fluorescence characteristics, the microgels of p(HT) with a degradable network can afford to provide long HT molecules to render excellent biomedical applications. Therefore, the hydrolytic degradation of p(HT) microgels prepared at 100×, 200×, and 300× was studied at pH 7.4 and 37.5 °C. Also, to determine the effect of pH on the degradation kinetics, the time-dependent degradation of p(HT) 300× at three different pH values, e.g., pH 5.4, 7.4, and 9, were studied and the corresponding results are shown in Figure 8a,b. In Figure 8a, the amount of HT degradation with time in mg/g shows the crosslinker dependence release profile, and, as expected, the lesser the amount of crosslinker used in the syntheses of p(HT) particles, the higher the amount of HT release. The highest degradation was observed for p(HT) microgels prepared at 100×. By choosing the appropriate ratio of crosslinker prolonged time of HT release as well as the extent of release, the amount can be controlled. For example, at the end of 357 h, 226.8 mg/g of HT was degraded from p(HT) microgel prepared at 100× whereas 49.9 mg/g of HT was degraded from p(HT) microgels prepared at 300× at the same degradation time. 

In Figure 8b, the degradation amounts at different pH values are given for p(HT) microgels prepared at 300×. The highest ratio of the crosslinked p(HT) particles is chosen to illustrate that even 300× crosslinked p(HT) microgels are hydrolytically degradable at different pHs. As can be seen from the figure, there is no significant difference observed between the degradation profiles of p(HT) microgels at pH 7.4 and 5.4, while the least amount of degradation occurred at pH 9. A 47.1 ± 4.5 mg/g was degraded at pH 5.4 in 78 h, and about 25.6 ± 2.1 mg/g HT was degraded from p(HT) microgel at the same time at pH 9, which also established the controlled HT release profile for medium pH dependence. 

To further assess the biomedical application perspective of p(HT) microgels, their antimicrobial potency against a Gram-negative bacterium, *E. coli,* and a Gram-positive bacterium, *S. aureus* was tested. *E. coli* is a profoundly common bacterium and can be easily transmitted by fecal–oral route and it is reported to cause severe conditions such as bacteremia, sepsis, inflammatory bowel diseases, urinary tract infections, and bloody diarrhea [32]. *S. aureus* is a major human pathogen causing osteoarticular, skin, soft tissue, and device-related infections [33]. After 24 h incubation of HT molecules and p(HT) microgels with these pathogens, the minimum inhibition concentration (MIC) and the minimum bactericidal concentration (MBC) values were determined via inoculation on nutrient broth and nutrient agar, and the results were summarized in Table 1.

In Table 1, the MIC and MBC values of HT for each bacterium were determined as a 2.5 mg/mL concentration. The MIC value of p(HT) against *E. coli* and *S. aureus* bacteria was determined as 5.0 mg/mL. The MBC value of p(HT) against *S. aureus* was 10.0 mg/mL. An antibiotic, gentamycin sulfate was used as a positive control, and the MIC and MBC value of gentamycin sulfate against *E*. *coli* bacteria was 0.007 mg/mL, and against *S. aureus* bacteria it was found to be 0.015 mg/mL. Although similar to HT molecules, p(HT) microgels have much higher MIC and MBC values against both microorganisms in comparison to the widely used antibiotic gentamycin, which is commonly used in the wide range of treatment of diseases caused by these microorganisms; the antibacterial potency of p(HT) microgels can confer a great avenue for the prophylaxis purpose of prevention from infections. Therefore, p(HT) microgels deliver great biomedical potential. 

## 4. Conclusions

Here, p(HT) microgels were successfully synthesized using GDE as a crosslinker via a microemulsion technique in the lecithin/isooctane medium for the first time in a single step at different X ratios. The sizes of p(HT) microgels varied from a few hundred nm to a few tens of micrometers with mostly about a few tens of micrometers. It was also found that p(HT) microgels retain the fluorescent properties as well as their antioxidant properties concerning HT molecules assessed in TPC and TFC tests. Moreover, the biocompatibility test on fibroblast L929 revealed that p(HT) microgels are much more biocompatible than HT molecules up to a 1000 µg/mL concentration signifying their biomedicinally important use. Furthermore, a comparative α-glucosidase enzyme inhibition study of HT and p(HT) microgel demonstrated the inhibition effectiveness of p(HT) microgels that is slightly lesser than the HT molecules and may have great potential in diabetes. Finally, the p(HT) microgels’ hydrolytic degradation prepared at different X as well as their variable controllable degradation profile at different pH media further foster the biomedical use of p(HT) microgel with tunable degradation. The added value of the inherently antioxidant, fluorescent, and antibacterial capability of p(HT) microgel augmented the value of this biomaterial to a value significantly higher than that of most conventional synthetic and natural polymers for in vivo applications. The HT molecule is a natural dye used to diagnose diseases, including various cancers. The therapeutic aspects of the prepared p(HT) microgels such as antimicrobial, antioxidant, and enzyme inhibiting capability with their inherent fluorescent property, good blood compatibility, and nontoxicity demonstrated in this study revealed the numerous biomedical potential applications of these microgels with tunable degradation capability. Therefore, the synthesized p(HT) microgels with these initial bio-beneficial in vitro results could be very useful for future in vivo studies in which both diagnosis and treatment can be possible as theragnostic materials due to retained innate properties of HT as well as the improved new properties of p(HT) upon particle formation as a viable material.

## Figures and Tables

**Table 1:** MIC and MBC values of HT and p(HT) microgels against *E. coli* ATCC 8739 and *S. aureus* ATCC 6538 after 24 h incubation *.

	*E. coli*		*S. aureus*	
Materials	MIC (mg/mL)	MBC (mg/mL)	MIC (mg/mL)	MBC (mg/mL)
HT	2.5	2.5	2.5	2.5
p(HT)	5	-	5	10

* Gentamycin sulfate was used as a positive control.

## Data Availability

The data presented in this study are available on request from the corresponding author.
